# Dercum’s Disease: Bridging Present Understanding and Emerging Directions

**DOI:** 10.3390/life16020290

**Published:** 2026-02-08

**Authors:** Francesca Gorini, Alessio Coi, Alice Verdelli, Elisabetta Magnaterra, Manfredi Magliulo, Luca Sanna, Virginia Corti, Simone Landini, Marta Donati, Irene Bonanni, Rachel Daher, Alberto Corrà, Cinzia Pupilli, Elena Biancamaria Mariotti, Valentina Ruffo di Calabria, Alessandro Magnatta, Marzia Caproni

**Affiliations:** 1 Unit of Epidemiology of Rare Diseases and Congenital Anomalies, Institute of Clinical Physiology, National Research Council, 56124 Pisa, Italy; francesca-gorini@cnr.it (F.G.); alessio.coi@cnr.it (A.C.); 2 1st Dermatological Clinic, Azienda USL Toscana Centro, P.O. Piero Palagi, Department of Health Science, University of Florence, 50122 Florence, Italy; alice.verdelli@uslcentro.toscana.it (A.V.); irene.bonanni@edu.unifi.it (I.B.); 3 Oncologic Dermatology Unit, IRCCS Azienda Ospedaliero-Universitaria di Bologna, 40126 Bologna, Italy; elisabetta.magnaterra@unibo.it; 4 Section of Dermatology, Department of Health Sciences, University of Florence, 50134 Florence, Italy; manfredi.magliulo@unifi.it (M.M.); luca.sanna@unifi.it (L.S.); simone.landini@unifi.it (S.L.); rachel.daher@unifi.it (R.D.); 5 Dermatology Department, University of Modena and Reggio Emilia, 41124 Modena, Italy; elettradonati@gmail.com; 6 Dermatology Unit, Ospedale San Bartolo, 36100 Vicenza, Italy; alberto.corra.92@gmail.com; 7 SOSD Endocrinology ASL Center, 50122 Tuscany, Italy; cinzia.pupilli@uslcentro.toscana.it; 8 Unit of Dermatology, Azienda USL Toscana Nord Ovest, 55100 Lucca, Italy; elenabiancamaria.mariotti@uslnordovest.toscana.it; 9 SSD Dermatology, Cardinal Massaia Hospital, 14100 Asti, Italy; valentina.ruffodicalabria@unifi.it

**Keywords:** Dercum’s disease, adiposis dolorosa, lipomatosis, rare disease, chronic pain, subcutaneous tissue, differential diagnoses, disease management

## Abstract

Dercum’s disease (DD) is a rare condition characterized by intense, asymmetrical, chronic burning pain localized in adipose tissue, often accompanied by subcutaneous fat nodules, leading to a significant reduction in quality of life. It typically affects overweight or obese adults between 35 and 50 years of age, with a marked female predominance. Despite numerous hypotheses proposed over time, the pathophysiology of DD remains poorly understood. Diagnosis is particularly challenging, as it relies solely on clinical evaluation. Given the overlapping features with other conditions, including symptoms, clinical course and inheritance pattern, a differential, accurate, and timely diagnosis is essential for the effective management of DD. Current treatment strategies focus primarily on pain relief, reflecting the still uncomplete understanding of DD etiopathogenesis. This review provides an updated overview of the current knowledge on DD, with particular emphasis on recent advances in pharmacological treatment strategies.

## 1. Dercum’s Disease: An Overview

Dercum’s disease (DD) (ORPHA:36397), also known as adiposis dolorosa, is a rare disease listed in Orphanet and in the National Organization of Rare Disorders [1,2]. The hallmark of DD is the progressive development of multiple lipomas within the adipose tissue [3]. Lipomas are typically located subcutaneously in the limbs and trunk, although they may also occur in deeper anatomical sites, where they are embedded within connective tissue and attached to muscle, bone, ligaments, or tendons, while generally sparing visceral sites [2]. In DD, the lipomas are associated with chronic pain lasting at least 3 months, which substantially impairs the quality of life (QoL) of patients [3,4]. The disease is frequently accompanied by a range of associated symptoms including overweight or obesity, fatigue and generalized weakness, alongside psychiatric manifestations such as anxiety, depression, and sleep disturbances [5,6,7]. Notably, although the disease is commonly seen in obese women, males with a normal body mass index (BMI) can also be affected [8].

The disease typically manifests between the ages of 35 and 50 and occurs 5 to 30 times more frequently in women than in men [7]. The early assumption that DD predominantly affects postmenopausal women, does not appear to be substantiated by robust evidence [7,9]. Interestingly, although DD usually appears in adulthood, it has also been sporadically described in pediatric age [10]. The exact prevalence of DD is currently unknown; however, estimates derived from various data sources suggest that fewer than 200,000 individuals are affected in the United States, thereby supporting its classification as a rare disease [11]. Notably, despite the predominantly sporadic occurrence of DD, a few case-reports have documented an autosomal dominant inheritance pattern, characterized by uncomplete penetrance and variable phenotypic expression [8,9].

### 1.1. Pathophysiology

**Figure 1:**
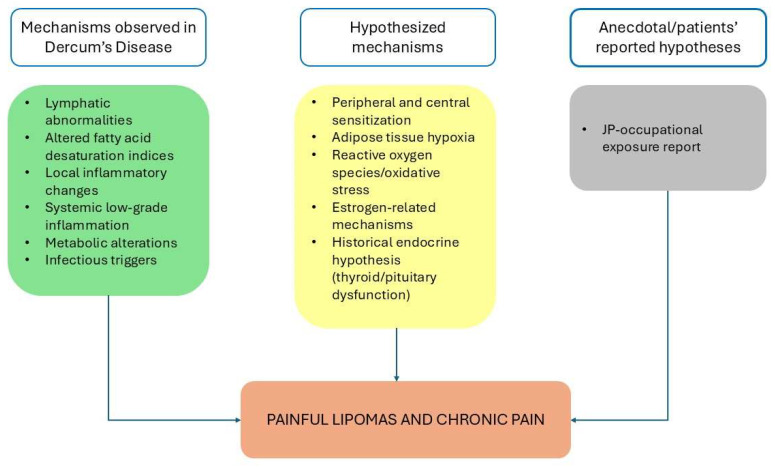
Pathophysiological hypotheses and levels of evidence in Dercum’s Disease.

Although first described in 1888, the pathophysiology of DD remains only partially understood [11]. A wide range of theories has been proposed to elucidate its etiology [3,7]. Some mechanisms have been observed in DD cohorts, including lymphatic abnormalities, distinct fatty-acid desaturation indices, isolated inflammatory findings, and selected metabolic alterations. In contrast, other proposed mechanisms are extrapolated from obesity-related biology and chronic-pain models, and have not yet been directly validated in DD (Figure 1).

DD was initially attributed to thyroid or pituitary dysfunction. An early survey of 100 individuals with DD reported a prevalence of approximately 27% of thyroid disorders, with hypothyroidism and subclinical hypothyroidism being the most common conditions [9]. More recently, Moattari et al. described a case of a woman with DD and a prior history of Hashimoto’s disease [6]. However, the majority of subsequent studies did not identify endocrine abnormalities in patients with DD [3,7].

In an effort to explain pain in DD, it has been further hypothesized that the sympathetic nervous system may form abnormal connections with peripheral sensory nerves, leading to the activation of pain fibers [7]. Accumulating evidence suggests that chronic pain primarily arises from inflammation within the peripheral nervous system (PNS) and central nervous system (CNS) [12]. During neuroinflammation, activation of glial cells (astrocytes, oligodendrocytes, microglia, satellite glial cells, Schwann cells) together with immune cells (macrophages, neutrophils) in the PNS leads to the release of inflammatory mediators such as prostaglandins, bradykinin, cytokines, and chemokines [12]. Upon binding to their respective receptors, these mediators induce hyperexcitability and hypersensitivity of nociceptive neurons (peripheral sensitization) through modulation of various ion channels, including transient receptor potential ion channels and voltage-gated sodium channels [12]. This cascade ultimately promotes the generation and exacerbation of pain [13]. In addition, pain may partly result from placebo responses or, alternatively, patients with DD may exhibit altered sympathetic activity as a consequence of pain [7]. Nonetheless, to date, no consistent evidence of neural inflammation, neural dysfunction, or structural nerve damage has been demonstrated in DD [7]. Painful nodules of adipose tissue have also been associated with rheumatoid arthritis [14] and traumatic injury [15].

An anecdotal hypothesis proposed by American military veterans, based on a single patient report, suggests that the development of multiple lipomas may be linked to occupational exposure to JP-8, a kerosene-based fuel commonly used in military aviation, given reports of DD onset or exacerbation among veterans during service [16]. JP-8 has been linked to long-term immunosuppressive effects in exposed individuals, with mice aerosolized with JP-8 for 1 h/day exhibiting immediate secretion IL-10 and prostaglandin E2, two immunosuppressive agents [17]. This immunosuppressive response was further associated with increased tumor metastasis in lung cancer models and reduced survival in experimental models of solid tumors [18]. Nonetheless, while these findings may suggest potential implications of JP-8 exposure, there is currently no scientific evidence supporting a causal relationship between JP-8 and the development of multiple lipomas in military veterans.

As previously mentioned, there is a strong association between obesity and DD, with reported obesity prevalence rates as high as approximately 73% [9]. In addition to substantially contributing to obesity, adipose tissue dysfunction may play a central role in DD pathophysiology [19]. When adipose tissue expands primarily through hypertrophy—an increase in the size of individual adipocytes—and exceeds its storage capacity, lipotoxicity ensues, leading to metabolic stress, inflammation, and impaired tissue homeostasis [20]. This condition is characterized by an excess circulating free fatty acids that accumulate in non-adipose organs (e.g., liver, muscle, heart, pancreas), altered secretion of adipokines, and systemic inflammation marked by elevated levels of interleukin (IL)-6, tumor necrosis factor-alpha (TNF-α), and C-reactive protein (CRP) [20,21]. Additionally, hypoxia within adipose tissue, resulting from the inability of the vasculature to keep pace with tissue growth, activates hypoxia-inducible factor 1 (HIF-1) [22]. HIF-1 serves a substrate for various kinase pathways, including phosphatidylinositol-3 kinase and the mitogen-activated protein kinase, extracellular signal-regulated kinase and p38, promoting fibrosis and contributing to angiogenesis, cell proliferation, apoptosis, dyslipidemia and inflammatory responses [23]. These processes ultimately lead to metabolic dysfunction, including reduced insulin sensitivity and glucose intolerance [20]. Furthermore, hypoxia partially inhibits mitochondrial electron transport, leading to increased production of reactive oxygen species (ROS), reduced antioxidant capacity, and impaired fat cell function and energy balance [20,23,24]. Estrogens may also play a role in DD pathogenesis, given several lines of evidence indicating their key role in regulating adipose tissue development and function in mammals [19,25]. A decline in estrogen levels, a condition typical of menopause in women, may lead to increased lipolysis, reduced insulin responsiveness, decreased serum leptin (an adipokine crucial for maintaining cellular energy homeostasis), enhanced recruitment of pro-inflammatory M1 macrophages, and elevated serum levels of pro-inflammatory cytokines such as TNF-α, IL-1β, and IL-6, supporting the relevant role of estrogens in the regulation of adipogenesis and adipose tissue activity, as well as in obesity-related dysfunction [25]. While the systemic inflammation observed in DD aligns with the pro-inflammatory state typically associated with the disease, and insulin resistance can be attributed to excessive fat accumulation [10,19] (see Section 1.3), other mechanisms—such as hypoxia-driven HIF-1 pathways, ROS production and the potential role of estrogens—can only be hypothesized based on adipose tissue physiology rather than on direct evidence obtained from DD patients.

Considering the close anatomical and functional relationship between lymphatic vessels and adipose tissue, and the established link between congenital lymphatic insufficiency and lymphedema, it is not surprising that, following an inflammatory process, patients with DD exhibit structural and functional alterations of the lymphatic system, ultimately leading to edema, fibrosis, and the formation of painful fatty masses [26,27]. Indeed, Rasmussen et al. [20] observed dilated, tortuous, and tender lymphatics in three women with DD, and their association with lipomas [27].

The lymphatic system is an immune organ and is therefore susceptible to infections [26]. Accordingly, over time various infectious agents have been proposed as potential causes of DD, including influenza, measles, herpes zoster, and malaria [26]. More recently, Beltran reported five cases of borreliosis, one of histoplasmosis, and one of coccidioidomycosis in seven women with DD, with infections occurring from less than one year to several years before disease onset [26]. Conversely, three of these patients developed lipomas early in life, potentially predisposing them to alterations in lymphatic drainage and, consequently, to an increased susceptibility to infections and further exacerbation of fatty masses [26].

Previous studies have put forward the hypothesis that DD may result from defects in lipid metabolism, a process known to contribute to the development of obesity due to the increased activity of stearoyl-CoA desaturase enzyme 1 (SCD1) [7,28]. SCD1 is the rate-limiting enzyme responsible for converting saturated fatty acids into delta-9 monounsaturated fatty acids, primarily producing oleate and palmitoleate from palmitate and stearate respectively, thereby enhancing hepatic de novo lipogenesis [29,30]. Yee et al. demonstrated that vaccenic/stearic desaturation index in subcutaneous adipose tissue (SAT) from participants with DD, was lower than that of obese controls, suggesting that SAT in DD displays distinct biochemical characteristics [28]. Given that the (palmitoleic + vaccenic)/palmitic acid ratio was decreased in these patients compared to obese controls and considering that vaccenic acid arises from chain elongation of palmitoleic acid rather than direct desaturation of stearate, it is plausible to speculate that DD involves a dysregulation in fatty acid desaturation, ultimately leading to impaired elongation to vaccenic acid [28]. Furthermore, subjects with DD showed a positive correlation between the palmitoleic/palmitic and oleic/stearic desaturation indices and both BMI and percent body fat, in alignment with experimental data [28,31].

### 1.2. Classification and Clinical Symptoms

Initially classified into three subtypes, DD is now clinically categorized into four distinct forms based on the distribution of the affected adipose tissue and its correlation with lipomas: type 1, the generalized diffuse form, characterized by widespread painful adipose tissue without clearly defined lipomas; type 2, the generalized nodular form, presenting with diffuse pain in the adipose tissue as well as in and around lipomas; type 3, the localized nodular form, in which pain is confined to lipomas and surrounding tissue; the type 4, the juxta-articular form, marked by solitary deposits of excess fat near joints [1,4,5]. A recent retrospective study involving 45 patients clinically diagnosed with DD, reported a prevalence of 51.1% of the localized nodular subtype [32]. While nodular forms are more easily identified, DD subtypes 1 and 2 are characterized by smaller and less detectable fat deposits [12]. Importantly, patients with DD subtype IV often experience reduced mobility of the knee joints and may develop gonarthrosis [33]. Moreover, as reported by Kucharz et al. [34], the juxta-articular form can coexist with the generalized form, resulting in a mixed phenotype.

As previously emphasized, DD is characterized by intense, asymmetrical, chronic (>3 months), burning pain localized in the adipose tissue, frequently accompanied by the presence of subcutaneous fat nodules and typically resistant to conventional analgesics [7,35,36]. In DD, multiple painful lipomas, which are highly variable in size, may develop in virtually any anatomical region [10]. These lesions most commonly occur on the trunk, accounting for nearly 70% of cases, and are also frequently observed near the limbs and buttocks [3]. In contrast, involvement of the head, neck, and breast is much rarer, yet clinically significant, as it may lead to severe occipital headache refractory to pharmacological treatment and mastalgia, respectively [37,38,39].

Unlike in earlier reports, asthenia and psychiatric manifestations are no longer considered cardinal symptoms of DD, although they may be associated with the condition in certain cases [4,7]. Asdourian et al. [32] reported that mood disorders and chronic fatigue syndrome were associated with 60% and 26.7% of DD cases, respectively. Notably, in fibromyalgia (FM), obesity is a common comorbidity that exacerbates disease severity and associated symptoms, including pain sensitivity, morning stiffness, sleep disturbances, physical strength, anxiety, and depression [40,41] (see Section 1.4). In addition to increasing the risk of rheumatic and musculoskeletal diseases, obesity exacerbates disease activity, pain, and fatigue, contributing to an overall decline in physical function [42]. Accumulating evidence also indicates that obesity and sleep disorders are closely interconnected through a bidirectional relationship involving inflammation, hormonal dysregulation, and autonomic dysfunction [43]. While sleep disturbances are associated with a range of cardiometabolic conditions, including obesity, arterial hypertension, dyslipidemia, insulin resistance, and type 2 diabetes (T2D), obesity predisposes to sleep disorders such as insomnia, obstructive sleep apnea, and restless legs syndrome, thereby amplifying cardiometabolic risk [43]. Elevated BMI, often driven by dietary patterns rich in saturated fats and sugars, contributes to metabolic and vascular dysfunction, including inflammation, insulin and leptin resistance, and hypertension (see Section 1.1), and may promote the development of anxiety and depression [43]. Indeed, alterations in brain nutrient composition can lead to neuroinflammation and potentially disrupt connectivity within corticolimbic networks that regulate mood, motivation, and emotional processing [44]. Chronic pain frequently coexists with a broad range of psychiatric disorders, including post-traumatic stress disorder, borderline personality disorder, and neuro-developmental conditions such as attention-deficit hyperactivity disorder [45]. Several mechanisms have been proposed to explain this comorbidity, e.g., genetic correlations, psychological and immune factors, alterations in bone-derived neurotrophic factor, dysregulation of the opioid and endocannabinoid systems, and dysfunction of the hypothalamic–pituitary–adrenal axis [45].

Other, albeit less specific, accompanying signs, such as digestive disorders (including constipation, bloating, and abdominal pain), shortness of breath, tachycardia, hypertension, diabetes, and endocrine disorders, have been described in case reports; however, there is currently no evidence supporting a definitive association with DD [1,3,46,47].

### 1.3. Other Parameters

In addition to abnormal fat accumulation, patients with DD may also present with metabolic and immunological alterations [3]. Izar et al. [10] described a prepubescent child with DD who exhibited insulin resistance, dyslipidemia, and low-grade systemic inflammation. Specifically, the case was characterized by hyperinsulinemia, reduced levels of high-density lipoprotein (HDL) cholesterol (an established marker of increased atherosclerotic cardiovascular risk, [48]), and a predominance of small, dense low-density lipoprotein (LDL) particles, which represent both a hallmark of obesity-related dyslipidemia [49] and a key driver of atherogenesis [50]. The patient also exhibited elevated levels of CRP and lipoprotein-associated phospholipase A_2_ (Lp-PLA_2_), an inflammatory biomarker of cardiovascular disease owing to its ability to hydrolyze oxidized phospholipids [10,51]. In circulation, Lp-PLA_2_ is bound to LDL cholesterol and shows a particularly high affinity for small, dense LDL particles [52]. Hypercholesterolemia has also been described in adults with DD, further reinforcing the association between adipose tissue dysfunction and metabolic impairment [53,54]. Regarding inflammation, Herbst et al. [55] found no significant difference in the number of macrophages within adipose tissue between women with and healthy controls. However, the presence of multi-nucleated giant cells (formed through the fusion of monocytes/macrophages, [56]) in 30% of DD patients may account for the significantly elevated levels of the pro-inflammatory cytokine IL-6 observed in these individuals. Meanwhile, the increased levels of IL-13 in women with DD may contribute to reduced concentrations of fractalkine (CX3CL1) and macrophage inflammatory protein (MIP)-1 beta, two chemokines involved, respectively, in inflammatory processes within the CNS through the promotion of macrophage chemotaxis and in the modulation of host responses at sites of acute and chronic inflammation, primarily via the recruitment of proinflammatory cells (monocytes, dendritic cells, and natural killer cells) [55,57,58]. IL-13 is a member of the T helper (Th)2 cytokine family and, in addition to playing a pivotal role in allergic inflammation and defense against parasite infection [59], it promotes immunoglobulin (Ig) switching to IgE and IgG4, stimulates B cell proliferation, and activates eosinophils, basophils, and mast cells [59]. Beyond these functions, IL-13 counteracts Th1-driven proinflammatory immune response [60]. Specifically, it contributes to a shift toward a more anti-inflammatory immunological profile by inhibiting several proinflammatory cytokines, including IL-8, MIP-3, monocyte chemotactic protein-3, and interferon-gamma-inducible protein 10 kDa [59]. Interestingly, although inflammatory markers such as CRP and erythrocyte sedimentation rate (ESR) can be elevated in DD [46,61], supporting the presence of an inflammatory response within the adipose tissue, the extent of this response does not appear significantly different from that observed in normal-weight controls [61]. Consistently, Mosbeh et al. [8] observed normal values for both CRP and ESR, as well as for blood cell counts, serum electrolytes, liver function tests, serum protein electrophoresis, and lipid profiles, confirming that inflammatory biomarkers lack specificity for DD and diagnosis is primarily determined through histological evaluation.

### 1.4. Differential Diagnoses

Diagnosis of DD is clinical and relies on a thorough physical examination and medical history, guided by the minimum criteria proposed by Hansson et al. [7]: the presence of painful lipomas that are tender on palpation in individuals with overweight or obesity, following the exclusion of other potential causal conditions [5,47,62]. Hansson’s minimum diagnostic criteria are the most widely cited in the literature and remain a useful clinical framework. While Dercum’s disease occurs primarily in overweight or obese individuals -particularly women- excess body weight should not be considered an absolute requirement for diagnosis. Indeed, although rarely, reports and cohort studies have documented DD in individuals with normal BMI, including males and pediatric patients [8,32]. Therefore, diagnosis should rely on the combination of clinical presentation and exclusion of alternative causes, rather than on body weight alone.

Ultrasound evaluation reveals the presence of multiple oblong lipomas with a hyperechoic appearance within the SAT, a feature that may help exclude malignancy [3]. Histological analysis can subsequently confirm the lipomatous and benign nature of the tissue [3]. Notably, Arsal et al. [35] recently reported a case involving subcutaneous nodules that appeared hyperechoic on ultrasound imaging, consistent with lipomas, but without any accompanying signs of metabolic dysfunction.

The main differential diagnoses to consider include lipedema, multiple symmetric lipomatosis (MLS), familial multiple lipomatosis (FML), FM, panniculitis, adipose tissue tumors, endocrine diseases, and psychiatric disorders [6] (Table 1). As misdiagnosis of DD is relatively common before a definitive diagnosis has been made, the true prevalence can be significantly underestimated [3,47].

- Although classified as a rare disease by some authors, lipedema has an estimated prevalence ranging from 11% to 39%, depending on the characteristics of the population studied [63]. Lipedema and DD are both severe, painful disorders of SAT, predominantly affecting women, and with a possible autosomal dominant inheritance with incomplete penetrance [46,64]. They are characterized by abnormal fat deposits that are typically resistant to reduction through starvation or excessive exercise [46]. Furthermore, lipedema is thought to be associated with dysregulated adipose estrogen, which may contribute to alterations to lipid accumulation, fatty acid uptake, and lipogenesis (see Section 1.1) [65]. Painful lipomas are absent in lipedema, and, unlike DD, lipedema is not closely associated with FM [3,46]. Although the distribution of SAT in lipedema and DD may appear similar, patients with lipedema exhibit a higher prevalence of nodules in the extracellular matrix and in the connective tissue surrounding the SAT areas in the lower body [46]. This pattern aligns with the characteristic gynoid distribution of lipedema, marked by a tendency to accumulate adipose tissue in the femoral-hip region [46,66]. In contrast to abdominal obesity, gynoid SAT seems to exert a protective role against T2D and cardiovascular disease, which could explain the lower prevalence of T2D observed in patients with lipedema compared to those with DD, despite the significantly higher BMI often reported in lipedema [46,67]. Lipedema can also be distinguished from DD through bioimpedance spectroscopy, which has revealed higher tissue water content in women with lipedema compared to those with DD [68]. Notably, lipedema and DD can also coexist, with some individuals exhibiting more affected SAT on the back, buttocks, and arms than typically seen in DD, and more pronounced involvement of the arms compared than in lipedema, suggesting that the two conditions may share pathophysiological features and, in some cases, progress toward one another [46].
- MSL, also known as Madelung disease or Launois–Bensaude lipomatosis, is a rare disorder of SAT, with an estimated prevalence of 1 in 25,000 (ORPHA:199276) [69,70]. It is characterized by the growth of symmetrical, painless, non-encapsulated masses of adipose tissue, predominantly around the face and neck [70]. This anatomical and symptomatic presentation distinguishes it from DD [47]. Additionally, contrary to DD, MSL primarily affects men, with a reported male-to-female ratio ranging from 15:1 to 30:1, typically manifesting between the third and the fifth decades of life [70,71]. Moreover, MSL is strongly associated with chronic alcohol consumption, predominantly in the form of red wine, with approximately 90% of patients reporting a history of moderate to heavy alcohol use [47,71,72]. Although the exact etiology and pathogenesis of MSL are still unclear, the condition is suspected to be linked to underlying metabolic disturbances, possibly involving functionally defective brown adipose tissue [72,73]. Classified into three phenotypes based on the distribution of adipose tissue, MSL typically occurs sporadically [71]. However, rare genetic forms have also been described [70]. Notably, mutations in mitochondrial tRNALys (a transfer RNA responsible for lysine incorporation during protein synthesis) such as the m8344A>G mutation; pathogenic variants in *MFN2*, a gene encoding a mitochondrial outer membrane protein involved in forming contact sites between mitochondria and the endoplasmic reticulum, which are important for calcium signaling and lipid metabolism; biallelic variants in the *LIPE* gene, which lead to the absence of hormone- sensitive lipase expression and consequently impairs adipocyte differentiation, have been associated with the MSL phenotype [5,62,73,74].
- FML is a rare adipose tissue disorder (ORPHA:199276, [75]) with a global incidence estimated at 0.002%, and no significant differences in prevalence between sexes [76,77]. Although FML typically manifests in the third decade of life, there are no strict age boundaries for onset, and it can also occur in children [77]. Encapsulated lipomas usually develop on the trunk and limbs, with involvement of the lumbar region, and, unlike DD, they are not associated with pain [77]. This condition follows an autosomal dominant pattern, although inheritance can vary considerably among affected individuals within the same family [76,77]. Certain genetic abnormalities have been identified as potentially contributing to the FML pathogenesis [76]. Among these, the high mobility group protein AT-hook 2 (*HMGA2*) encodes a non-histones chromatin protein that acts as a transcriptional factor, regulating the gene expression program during adipogenesis [77,78]. Overexpression of *HMGA2* has been linked to obesity and its aberrant expression has also been documented in lipomas and in a variety of adipocytic tumors [70,79].
- FM is a complex chronic disorder characterized by widespread musculoskeletal pain (lasting at least 3 months, like in DD) and recurrent episodes of intense fatigue, often accompanied by cognitive difficulties, sleep disturbances, and psychiatric symptoms such as anxiety and depression, all occurring in the absence of a clearly identifiable pathological cause [2,3,80]. The most affected body regions include the back of the neck, shoulders, lower back, elbows, hips, and knees [2]. Furthermore, while pain in FM is initially localized, it tends to progress over time, affecting multiple muscle groups and often manifesting as a burning sensation and with eventually concomitance of hyperalgesia (increased pain in response to normally painful stimuli, such as pressure or heat) and allodynia (pain in response to normally non-painful stimuli, such as light touch, or temperature variations) [81]. For these reasons, FM greatly impairs QoL of affected individuals [82]. FM has a global prevalence estimated at approximately 5% with a marked female predominance, accounting for 75–90% of all cases, and peak incidence occurring between 20 and 50 years of age [81,83]. If on the one hand FM is a disorder of pain regulation, primarily driven by abnormal sensory processing within the CNS, on the other side genome-wide association studies also suggest a role of genetic predisposition in its pathogenesis [84]. Among the candidate genes implicated in FM is the serotonin transporter gene (*SLC64A4*), whose S allele variant has been associated with various conditions, including depression, schizophrenia, anxiety disorder, and chronic pain [85]. Notably, a recent systematic review and meta-analysis revealed that obesity is highly prevalent among patients with FM and exacerbates several aspects of the disease including pain, stiffness, fatigue, impaired physical functioning, sleep disturbances, and cognitive dysfunction [82].
- Panniculitis, a relatively uncommon inflammation of SAT, is associated with a wide spectrum of inflammatory diseases that are traditionally regarded among the most diagnostically challenging for both clinicians and pathologists [86,87]. Clinically, it usually presents with painful nodules or plaques, which may represent the initial manifestation of underlying dermatologic or rheumatologic diseases [86]. Accurate diagnosis often requires biopsies of adequate sampled lesions and histopathological evaluation allows classification of panniculitis into septal or lobular inflammatory patterns, with or without associated vasculitis [86,87]. Erythema nodosum (EN), the most prevalent type of panniculitis in the setting of immune-mediated and autoimmune diseases, is also caused by infectious agents, drugs, and vaccination [86,87]. Despite variations across ethnic groups and geographical regions, the global incidence of EN is estimated at approximately 5 per 100,000 individuals [87]. EN typically manifests between the second and the fourth decade of life, with peak incidence occurring between 20 and 30 years and a female-to-male ratio ranging from 3:1 to 6:1 [87]. In EN, there is an acute development of painful, tender, erythematous, and warm nodules from 1 to 10 cm in diameter, generally located on the lower limbs, which can be accompanied by systemic symptoms such as low-grade fever, cough, and abdominal pain [87].
- Atypical lipomatous tumors (ALT), also known as well-differentiated liposarcomas (WDLPSs), account for 40–45% cases of all liposarcomas, which collectively represent the most common type of soft tissue neoplasms [88,89]. ALT/WDLPS predominantly affect middle-aged adults, with peak incidence between the fourth and fifth decades of life [88]. These tumors most frequently present as slowly enlarging, painless masses in deep soft tissue of proximal extremities and retroperitoneum and, although tend to recur locally with a rate of approximately 30–50%, they lack metastatic potential [88,90]. Nonetheless, ALT/WDLPSs have the capacity for differentiation, occurring in approximately 10% of cases, with this risk increasing in deep-seated tumors, particularly those arising in the retroperitoneum, and following surgical resection [90,91]. Like their dedifferentiated counterparts, ALT/WDLPSs contain high-level amplifications of chromosome 12q13–15 [90]. This chromosomal region encompasses several genes including *MDM2*, whose overexpression is considered a key event in ALT/WDLPS pathogenesis [90]. Indeed, *MDM2* binds to p53, inhibiting its function and thereby preventing apoptosis and promoting tumor proliferation [90]. As benign lipomas cannot always be reliably distinguished from ALT/WDLPS through solely histological examination, pathognomonic 12q13-15 amplifications resulting *MDM2* overexpression is considered the diagnostic gold standard [92].
- Among endocrine disorders, Cushing syndrome (CS) is a rare condition (ORPHA:96253, [93]), most commonly caused by adrenocorticotropic hormone secreting pituitary tumors, which account for approximately 70–80% of cases [94,95,96]. CS is characterized by chronic hypercortisolemia and a heterogeneous clinical presentation, ranging from subtle to severe manifestation [97]. The estimated prevalence of CS is 57–79 cases per million individuals, with an incidence of 1.8–4.5 cases per million [96]. The condition predominantly affects women, with a female-to-male ratio of 4:1, and the mean age at diagnosis is approximately 44 years [96]. Weight gain is one of the hallmark features of CS, occurring in more than half of affected individuals [98]. In particular, chronic hypercortisolism is associated with preferential visceral—rather than subcutaneous—fat accumulation in the abdominal region, driven by abnormal adipokine production, a mechanism implicated in the development of metabolic syndrome [95,97]. Notably, visceral adipose tissue in patients with CS exhibits distinct characteristics, including enlarged abdominal adipocytes and enhanced lipogenic activity compared to obese individuals without CS [98].
- Multiple endocrine neoplasia type 1 (MEN1) is a rare hereditary syndrome (ORPHA:652) with an estimated global prevalence of approximately 1–20 per 100,000 individuals [99,100]. The condition affects males and females equally [99]. MEN1 occurs predominantly in familial form, with autosomal dominant inheritance in approximately 90% of cases [101]. It is caused by mutations in the *MEN1* gene, which encodes menin, a key regulator of cell proliferation and differentiation [100]. Affected subjects may exhibit more 20 endocrine and non-endocrine manifestations, among which primary hyperparathyroidism is the most common, being detectable in nearly all patients by the fifth decade of life [101]. Tumors in MEN1 can develop at any age, with parathyroid tumors being the most common, occurring in approximately 95% of cases, followed by pancreatic tumors and anterior pituitary adenomas (with prevalences of around 30–70% and 30–40%, respectively) [101]. In addition, the presence of cutaneous lesions such as angiofibromas, collagenomas, and lipomas is included in the diagnostic criteria for MEN1 [102]. Specifically, the presence of two or more of these lesions is considered diagnostic, while a single lesion is considered sufficient in individuals with a positive family history of MEN1 [103]. Lipomas, in particular, are observed in approximately 34% of patients and are predominantly located on the trunk, extremities, and scalp [102].
- Psychiatric disorders are frequently accompanied by chronic pain manifestations [104]. The prevalence of pain in psychiatric conditions can vary widely depending on pain type and anatomical site, with musculoskeletal pain reported in up to 93% of patients with anxiety and/or depression [104,105]. Conversely, individuals affected by chronic musculoskeletal pain are predominant in women and exhibit a significantly higher risk of developing psychiatric disorders compared to healthy controls, particularly anxiety disorders with panic attacks (44% of cases), generalized anxiety (36%), and mixed anxiety and depression disorder (33%) [106]. Therefore, chronic pain and anxiety/depression disorders are linked by a bidirectional relationship, which may be partly explained by the progressive social isolation induced by pain, which leads to the worsening of depression symptoms, and by the amplification of pain perception in individuals affected by depression [105].

**Table 1:** Overview of the principal differential diagnoses in Dercum’s disease.

Disease	Overlapping Aspects	References	Differences	References
Lipedema	Absence of lipomas (consistent with type 1 DD) Predominant in women Possible autosomal inheritance Possible co-existence with DD	[46,64]	Symmetrical disorder Not closely associated with FM Higher prevalence of nodular SAT areas in the lower body Higher tissue water content	[3,46,63]
MSL	SAT disorder	[70,71]	Symmetrical, painless non-encapsulated lipomatous deposits	[5,47,62,70,71,72]
	Occurrence between the third and fifth decades of life		Lipomas primarily located around the neck and face	
			More prevalent in men	
			Markedly linked to alcohol abuse	
			Possibility of 8344A to G mitochondrial mutation	
FML	Possibility of occurrence in children	[77]	No significant differences in sex prevalence Encapsulated painless lipomas occurring in the trunk, lower back and limbs	[77,78]
			Occurrence in the third decade of life	
			Autosomal inheritance pattern	
FM	Pain persisting for at least 3 months Associated symptoms: sleep disturbances, anxiety, depression	[2,3,80]	Absence of lipomas Widespread musculoskeletal pain with progressive course	[81]
Panniculitis (EN)	Painful nodules or plaques Higher prevalence in women	[86,87]	Nodules generally located on the lower limbs	[87]
			Possibility of concurrent systemic symptoms including low-grade fever, cough, and abdominal pain	
ALT/WDLPS	Peak incidence between the fourth and fifth decades of life	[88]	Painless masses of deep soft tissues of proximal extremities and retroperitoneum Pathogenesis linked to amplification and overexpression in genes mapped to chromosome 12q13–15	[88,90,91,92]
Cushing syndrome	Predominance in women	[96]	Chronic hypercortisolemia Visceral fat accumulation in the abdominal region	[96]
MEN1	Lipomas detected in 34% of cases predominantly located on the trunk and limbs	[102]	No differences in prevalence by sex Autosomal dominant inheritance	[98,99,101]
			Presence of painless lipomas in one third of patients	
Psychiatric disorders	Predominance in women Chronic pain Co-existence with DD	[104,105,106]	Absence of objective disease	[3]

Abbreviations: ALT: atypical lipomatous tumor; DD: Dercum’s disease; EN: erythema nodosum; FM: fibromyalgia; FML: familial multiple lipomatosis; MEN1: Multiple endocrine neoplasia type 1; MSL: multiple symmetric lipomatosis; SAT: subcutaneous adipose tissue; WDLPS: well-differentiated liposarcomas.

## 2. Management of Dercum’s Disease

As the etiopathogenesis of DD remains not fully clarified, no gold standard therapy has been established, and management is generally individualized and almost exclusively focused on pain relief through oral medication, direct injections and surgical excision [6,34,107]. Additionally, psychotherapy may serve as a valuable approach to help patients cope with this condition and mitigate external stressors that could worsen pain symptoms [3,34].

### 2.1. Surgical Interventions

Surgical options, which represent the first-line intervention in DD, include liposuction or lipectomy [33,108]. Liposuction, a safe procedure with a low risk of complications, is effective in removing localized excess adipose tissue [109] and may alleviate pain in DD patients [6]. On the other hand, it is less effective in treating long-standing juxta-articular type due to progressive fibrosis, for which lipectomy is generally preferred [33]. Wollina et al. [33] documented the successful treatment of four patients with type 4 DD, some of whom also had comorbidities such as hypertension, T2D, and cataracts. Following lipectomy, these patients experienced significant pain reduction within a few weeks and improved mobility, even at an advanced age [33].

In the study by Hanssen et al. [110], which evaluated the effect of liposuction on pain in 53 women with DD, both subjective and objective measurements demonstrated a postoperatively reduction in pain compared to preoperative levels, with sustained pain relief lasting for at least 5 years. As outlined in Section 1.1, pain in DD might arise from the dysfunction of sympathetic nervous system, in particular from aberrant signals transmitted to the spinal cord via abnormal connections between peripheral autonomic and sensory nerves [110]. Since nerve injury is one of the known effects of liposuction, it can be assumed that the procedure causes damage to a fraction of both sensory nerves and abnormal connections [110,111]. This may explain the only partial relief of pain observed in DD patients after liposuction [110]. Furthermore, the potential formation of new abnormal connections over time could account for the pain relapse experienced by DD patients several years postoperatively [110].

Recently, Young et al. [112] described the case of a patient who underwent two separate surgeries for the excision of a total of 35 lipomas distributed throughout the body. Two years later, the patient developed numerous new lipomas, which were again surgically removed [112]. Despite multiple interventions, both the lipomas and associated pain recurred, ultimately resulting in a substantial deterioration in QoL [112]. Therefore, if liposuction seems a valid approach to reduce pain in DD although with a relatively short duration, with no related complications, surgical excision cannot be employed in subjects continually developing widespread lesions [113,114].

### 2.2. Pharmacological Treatments

There are no specific pharmacological treatments for DD; clinical management focuses on reducing pain and improving the patient’s QoL through a symptomatic and multidisciplinary approach. Treatments should be tailored to each individual’s specific symptoms, with the primary goal of achieving pain relief.

Pharmacological management of DD is complex and often unsuccessful with standard treatments, typically focusing on analgesics [5]. Patients with DD are considered refractory to non-steroidal anti-inflammatory drugs (NSAIDs) [7], although some authors reported potential pain reduction with NSAIDs. Specifically, Herbst et al. [9] reported pain relief in 89% of patients treated with NSAIDs and in 97% of those receiving narcotic analgesics. However, chronic NSAID therapy is generally discouraged because long-term use is associated with a substantially increased risk of gastrointestinal complications, including peptic ulcer disease and bleeding, as well as other systemic adverse effects. Other authors have proposed paracetamol as a first-line treatment, with opioids representing an effective option for patients experiencing severe pain [115,116].

Intravenous administration of lidocaine (ATC code: N01BB02) can provide patients with sustained pain relief lasting weeks to months, whereas direct injections of lidocaine into painful lipomas are less effective, typically offering only transient benefit [117,118,119]. The exact mechanism of action of lidocaine is not fully understood, but animal studies suggest that it may involve sodium channel modulation in peripheral nerves or increased sympathetic nervous system activity [7,118]. Another anesthetic, mexiletine (ATC code: C01BB02), has been successfully used as an alternative or adjunct to lidocaine and can also be administered orally in tablet form [120].

The use of corticosteroids (CCS) has also been described in earlier studies; however, clinical outcomes remain inconsistent and controversial [7]. Some authors have documented improvement in patients treated with prednisolone [121,122], whereas Weinberger et al. reported significant amelioration in two cases following intralesional methylprednisolone injections [123]. The proposed mechanism of action involves inhibition of mediators such as histamine, serotonin, bradykinin, and prostaglandins, given the likely inflammatory etiology of DD (see Section 1.1) [124]. Conversely, other reports have associated CCS administration with worsening of pain symptoms [125].

Beyond NSAIDs, anesthetics, and CCS, numerous other medications have been tested—often anecdotally and in isolated cases—reflecting the heterogeneous nature of the disease. These include methotrexate (MTX), infliximab (IFX), glucagon-like peptide-1 (GLP-1) receptor agonists such as semaglutide (SEMA) and tirzepatide (TRZ), calcium channel modulators (e.g., pregabalin), interferon α-2b, metformin, d-amphetamine, and deoxycholic acid.

MTX (ATC code: L04AX03) is an antimetabolite antifolate widely employed as an immunosuppressant in autoimmune diseases (at low doses) and as a chemotherapeutic agent (at high doses) [126]. Its anti-inflammatory effect at low doses is primarily mediated through inhibition of 5-aminoimidazole-4-carboxamide ribonucleotide (AICAR) transformylase, leading to AICAR accumulation and a subsequent extracellular increase in adenosine [127]. Adenosine exerts anti-inflammatory actions by suppressing immune cell activation and reducing the release of pro-inflammatory cytokines [127]. MTX further modulates cytokine production and immune cell function—including T cells, B cells, and macrophages—while decreasing pro-inflammatory mediators such as TNF-α, IL-1, and IL-6 across diverse inflammatory contexts [128]. Evidence regarding MTX use in DD is extremely limited and largely based on case reports and small case series. Singal et al. reported symptomatic improvement, including pain reduction, weight loss, and enhanced metabolic parameters, in a patient treated with MTX and IFX [129]. Similar findings were subsequently observed in a woman with DD who experienced pain relief and a reduction in forearm lipoma size following combined MTX and IFX therapy [107].

IFX (ATC code: L04AB02) is a chimeric monoclonal antibody targeting TNF-α, a cytokine that plays a key role in sustaining chronic mucosal inflammation [130]. IFX binds to both soluble and membrane-bound TNF-α, neutralizing its activity by preventing interaction with cellular receptors [130]. Additionally, IFX modulates immune cell function by inducing apoptosis of activated cells, regulating macrophages, and reducing the production of pro-inflammatory cytokines [130].

Authors reporting improvement in DD with combined IFX and MTX therapy have underscored that it remains unclear whether the observed benefit was attributable solely to IFX or whether MTX exerted an additive effect [129]. Considering DD as a potential autoimmune disorder, the anti-inflammatory properties of IFX, MTX, or both may contribute to clinical improvement. Specifically, IFX, as a TNF-α inhibitor, may ameliorate DD by suppressing TNF-α–mediated release of circulating free fatty acids, a mechanism described by Ryden et al. [131], thereby reducing peripheral insulin resistance. Indeed, as described in Section 1.1., adipose tissue dysfunction, through cytokine secretion, perpetuates systemic low-grade inflammation, which in turn promotes ectopic fat deposition, metabolic dysregulation, ultimately leading to multi-organ damage [12].

Magnatta et al. reported the use of a triple combination therapy consisting of MTX, IFX, and SEMA (ATC code: A10BJ06), a GLP-1 receptor agonist, achieving an ongoing 9-month remission in one patient, with reductions in DLQI, VAS, and BMI [132]. SEMA activates GLP-1 receptors both centrally and peripherally, enhancing glucose-dependent insulin secretion, delaying gastric emptying, and reducing appetite; at doses used for obesity, it induces significant weight loss [132]. Although direct clinical evidence on SEMA use in DD is limited, its mechanism of action provides a plausible rationale for therapeutic benefit. Specifically, by promoting weight loss, SEMA may reduce total adipose tissue mass in obese DD patients, thereby improving metabolically mediated inflammation [133]. Furthermore, preclinical and review studies suggest that GLP-1 receptor activation can attenuate systemic inflammation and modulate the inflammatory profile of adipose tissue [134,135]. Given that an inflammatory component within the panniculus and fat lesions has been documented in some DD patients, the ability of SEMA and other GLP-1 receptor agonists to modulate adipose inflammation represents a potential mechanism of action.

In the previously cited Italian study, concomitant administration of MTX and TRZ (ATC code: A10BX16)—a dual glucose-dependent insulinotropic polypeptide-GIP/GLP-1 receptor agonist that activates both GLP-1 and GIP receptors—successfully induced ongoing remission in one patient, with reductions in DLQI, VAS, and BMI persisting for more than nine months [132]. Conversely, in another patient, the same regimen yielded only minimal clinical benefit and was discontinued two months after TRZ initiation due to malaise [132].

Calcium-channel modulators, such as pregabalin (ATC code: N02BF02), have also been explored as therapeutic options. Lange et al. reported the case of a patient who responded successfully to pregabalin combined with manual lymphatic drainage [118]. Pregabalin is commonly used to manage neuropathic pain by binding to neuronal calcium channels, thereby inhibiting the release of excitatory neurotransmitters such as amino acids, which play a key role in central sensitization [136].

Single reports on beneficial effects of interferon α-2b, metformin and d-amphetamine were also published. Gonciarz et al. reported long-term pain relief in two patients with DD treated with interferon α-2b [137]. The authors proposed several mechanisms underlying this improvement: (i) the drug’s antiviral properties; (ii) stimulation of endorphin production; and (iii) interference with the synthesis of IL-1 and tumor necrosis factor-alpha (TNF-α, both of which are implicated in cutaneous hyperalgesia [137].

Łabuzek et al. documented a case of DD treated with metformin (ATC code: A10BA02), a drug primarily used in obese patients with T2D, observing effects of metformin on adipokines, β-endorphin, and pro-inflammatory cytokines, which were associated with pain relief [138]. The hypothesized mechanisms of underlying metformin-induced pain reduction may involve metabolic pathways as well as additional actions, such as modulation of synaptic plasticity and activation of microglia [138].

Ghazala et al. described two cases of patients DD who demonstrated clinical improvement after several months of treatment with low-dose d-amphetamine, which was also associated with regression of hepatic fat deposits [139]. The authors hypothesized that these effects may be mediated through enhanced lipolysis, sympathetic nervous system activation, and metabolic modulation [139].

Recently, deoxycholic acid (DCA; ATC code: D11AX24) has been employed in the management of DD. DCA is a secondary bile acid that induces adipocyte lysis when injected into subcutaneous fat tissue [140]. Wipf et al. reported the effective use of DCA injections for managing lipomas in DD patients [141]. In 2021, Rice et al. documented intralesional DCA administration to reduce pain and lipoma size, confirming lipoma reduction through radiographic evidence [114]. More recently, Schwartz et al. suggested that intralesional DCA injections represent a safe and effective nonsurgical alternative for patients with multiple lipomas where surgical intervention is not feasible or desired [108]. They described a DD patient with worsening pain and multiple lipomas who achieved pain reduction and improved mobility after three rounds of DCA injections [108].

In conclusion, the discrepancy between the reported ineffectiveness of certain drugs and success in specific cases may reflect the existence of DD subtypes, each responding differently to therapy (Table 2). This clinical heterogeneity complicates the establishment of standardized treatment protocols and underscores the need for individualized therapeutic strategies.

### 2.3. Other Treatments

Initially employed in a single patient with generalized diffuse DD], treatment with the transcutaneous frequency rhythmic electrical modulation system (FREMS), a sequence of software-driven modulated electrical stimuli that vary automatically in pulse frequency, duration, and voltage amplitude, has emerged as an effective and safe therapeutic option [142]. Following 4 cycles of 30 min sessions over a 6-month period, the patient exhibited significant improvement in clinical signs and symptoms, including a marked reduction in pain (from 64 to 17 points in the VAS scale), enhanced daily abilities (evaluated through Barthel index), and overall health status (Short Form-36 questionnaire) [142]. These clinical benefits were accompanied by a concurrent decrease in total fat body mass (approximately 7 kg) and SAT thickness at the abdominal wall (1.2 cm) [142]. In a subsequent study, seven patients with type II generalized diffuse form of DD underwent five cycles of FREMS therapy administered over a one-year period, with sessions scheduled at baseline and at 3, 6, 9, and 12 months [62]. Consistently with [134], VAS scores decreased, although at the limit of statistical level, during the study period, and certain SF-36 domains, i.e., physical functioning, role limitation due to physical health, body pain, vitality, social functioning, showed a significant improvement, underscoring the potential of FREMS therapy to alleviate pain and ameliorate QoL [62]. FREMS has been successfully employed in the treatment of painful diabetic neuropathy [143], myofascial pain syndrome [144], chronic and painful venous leg ulcers [145], and, as reported in a case report, in scleredema diabeticorum [146]. Although its mechanism of action is only partially understood, FREMS may exert therapeutic effects by overcoming the skin’s dielectric barrier and recruiting membrane potentials in excitable tissues [143]. FREMS has been shown to enhance microvascular blood flow and vasomotor activity, and to promote the release of vascular endothelial growth factor, thereby contributing to improved nerve health and a reduced perception of pain [62,143]. On the other hand, patients with DD undergoing FREMS, generally assume analgesic drugs, to cope with pain, therefore it is difficult to determine whether the reduction in pain is exclusively attributable to FREMS [62].

## 3. Conclusions

Dercum’s disease is a rare, chronic, and debilitating condition characterized by painful SAT growth, with an etiology that remains partly unknown. Although general diagnostic protocols for DD include a thorough clinical history, physical examination, assessment of serologic acute-phase inflammatory markers, and biopsy of suspicious lesions, diagnosis remains challenging due to the disease’s rarity and heterogeneous clinical presentation. DD typically manifests as multiple benign lipomas accompanied by nonspecific symptoms such as pain, fatigue, and neuropsychiatric disturbances, which often overlap with other conditions and complicate differential diagnosis. These challenges highlight the urgent need for definitive and universally accepted diagnostic criteria to improve recognition and management. Currently, no standardized pharmacological regimen exists, and robust evidence supporting drug efficacy is lacking. Notably, the current therapeutic evidence for DD is based almost exclusively on isolated case reports and small case series, with no controlled clinical trials available to date. Therefore, further systematic and controlled studies are warranted to establish evidence-based therapeutic strategies. In localized forms, surgical reduction in fatty lesions may alleviate symptoms; however, treatment should be individualized according to clinical subtype and symptom severity. Additional research is essential to elucidate DD’s etiopathogenesis and identify potential risk factors, both of which are critical for developing targeted and effective therapeutic strategies aimed at enabling earlier intervention and improving patient QoL.

## Figures and Tables

**Table 2:** Pharmacological treatments in Dercum’s disease: key features.

Medication	Mechanisms of Action	Main Effects	References
NSAIDs	Inhibition of inflammatory mediators	Pain relief	[7,9]
Lidocaine and/or mexiletine	Sodium channel blockade and consequent inhibition of peripheral nerve conduction	Sustained pain relief	[117,118,119,120]
CSS	Anti-inflammatory effects	Mixed outcomes: clinical improvement in some cases, risk of pain worsening.	[121,122,123,124,125]
MTX	Inhibition of AICAR transformylase; immunomodulation of T/B cells, and macrophages	Improvements in BMI, DLQI, VAS, and metabolic parameters; pain relief.	[107,128,129,130]
MTX + IFX	Inhibition of AICAR transformylase; immunomodulation of T/B cells, and macrophages; blockade of pro-inflammatory signaling. Overall, synergistic immunomodulation	Documented 15-month remission	[120,131,132]
MTX + IFX + SEMA	Inhibition of AICAR transformylase; immunomodulation of T/B cells, and macrophages; blockade of pro-inflammatory signaling; activation of GLP1-1 receptors, with consequent appetite decrease and attenuation of systemic inflammation	Reductions in DLQI, VAS, and BMI	[129,133,134,135]
MTX + TRZ	Inhibition of AICAR transformylase; immunomodulation of T/B cells, and macrophages; activation of GLP-1 and GIP receptors	Reductions in DLQI, VAS, and BMI variable and not consistent across patients	[129]
Pregabalin	Calcium-channel modulation in CNS with consequent reduction of excitatory neurotransmitter release	Pain relief	[118]
Interferon α-2b	Antiviral activity; stimulation of endorphin production; immunomodulation of cytokine release	Long-term pain relief	[137]
Metformin	Promotion of favorable profiles of adipokine, β-endorphin, and pro-inflammatory cytokine; modulation of synaptic plasticity; activation of microglia	Pain relief	[138]
d-amphetamine	Enhancement of lipolysis; activation of sympathetic nervous system	Clinical improvement with decrease in hepatic fat deposits	[139]
DCA	Adipocyte lysis	Reduction in lipoma size and pain; improved mobility	[108,114,140,141]

Abbreviations. AICAR: 5-aminoimidazole-4-carboxamide ribonucleotide; BMI: body mass index; CNS: central nervous system; CSS: corticosteroids; DCA: deoxycholic acid; DLQI: Dermatology Life Quality Index; GIP: glucose-dependent insulinotropic polypeptide; GLP-1: glucagon-like peptide 1; IFX: infliximab; MTX: methotrexate; NSAID: non-steroidal anti-inflammatory drug; SEMA: semaglutide; TRZ: tirzepatide; VAS: Visual Analogue Scale.

## Data Availability

No new data was created.

## Abbreviations

The following abbreviations are used in this manuscript:

ALT	Atypical lipomatous tumor
BMI	Body mass index
CCS	Corticosteroids
CNS	Central nervous system
CS	Cushing syndrome
DCA	Deoxycholic acid
DD	Dercum’s disease
DLQI	Dermatology Life Quality Index
ESR	Erythrocyte sedimentation rate
FM	Fibromyalgia
FML	Familial multiple lipomatosis
FREMS	Frequency rhythmic electrical modulation system
GIP	Glucose-dependent insulinotropic polypeptide
GLP-1	Glucagon-like peptide-1
IFX	Infliximab
IL	Interleukin
MEN1	Multiple endocrine neoplasia type 1
MIP	Macrophage inflammatory protein
MLS	Multiple symmetric lipomatosis
MTX	Methotrexate
NSAID	Non-steroidal anti-inflammatory drug
PNS	Peripheral nervous system
QoL	Quality of life
SAT	Subcutaneous adipose tissue
SCD1	Stearoyl-CoA desaturase enzyme 1
SEMA	Semaglutide
T2D	Type 2 diabetes
TNF-α	Tumor necrosis factor-alpha
TRZ	Tirzepatide
VAS	Visual Analogue Scale
WDLPS	Well-differentiated liposarcoma
